# Evaluation of pharmacotherapy complexity in residents of long-term care facilities: a cross-sectional descriptive study

**DOI:** 10.1186/s40360-017-0164-3

**Published:** 2017-07-25

**Authors:** Vanessa Alves-Conceição, Daniel Tenório da Silva, Vanessa Lima de Santana, Edileide Guimarães dos Santos, Lincoln Marques Cavalcante Santos, Divaldo Pereira de Lyra

**Affiliations:** 1 Laboratory of Teaching and Research in Social Pharmacy (LEPFS), Federal University of Sergipe, São Cristóvão, SE, Brazil; Cidade Universitária “Prof. José Aloísio Campos”, Jardim Rosa Elze, São Cristóvão, CEP: 49100-000 Brazil; 2 Group of Studies in Geriatrics and Gerontology, College of Pharmacy, Federal University of Vale do São Francisco, Petrolina, Brazil

**Keywords:** Long-term care facilities, Elderly, Complexity of pharmacotherapy

## Abstract

**Background:**

Polypharmacy is a reality in long-term care facilities. However, number of medications used by the patient should not be the only predictor of a complex pharmacotherapy. Although the level of complexity of pharmacotherapy is considered an important factor that may lead to side effects, there are few studies in this field. The aim of this study was to evaluate the complexity of pharmacotherapy in residents of three long-term care facilities.

**Methods:**

A cross-sectional study was performed to evaluate the complexity of pharmacotherapy using the protocols laid out in the Medication Regimen Complexity Index instrument in three long-term care facilities in northeastern Brazil. As a secondary result, potential drug interactions, potentially inappropriate medications, medication duplication, and polypharmacy were evaluated. After the assessment, the association among these variables and the Medication Regimen Complexity Index was performed.

**Results:**

In this study, there was a higher prevalence of women (64.4%) with a high mean age among the study population of 81.8 (±9.7) years. The complexity of pharmacotherapy obtained a mean of 15.1 points (±9.8), with a minimum of 2 and a maximum of 59. The highest levels of complexity were associated with dose frequency, with a mean of 5.5 (±3.6), followed by additional instructions of use averaging 4.9 (±3.7) and by the dosage forms averaging 4.6 (±3.0).

**Conclusions:**

The present study evaluated some factors that complicate the pharmacotherapy of geriatric patients. Although polypharmacy was implicated as a factor directly related to complexity, other indicators such as drug interactions, potentially inappropriate medications, and therapeutic duplication can also make the use of pharmacotherapy in such patients more difficult.

## Background

Over the last few decades, the widespread use of drugs among individuals older than 65 years has been a subject of concern for health systems worldwide [1–3]. The geriatric population presents a challenge to the clinician due to the physiological changes specific of the aging process, including impaired renal and hepatic function (which has implications for drug metabolism and therefore, dosing), reduction in cognitive function, increased weakness, and loss of autonomy to perform basic daily activities compared to young and healthy adults. Furthermore, some factors increase the medication regimen complexity and the probability of drug-related problems [4, 5].

One of the most important aspects influencing the complexity of pharmacotherapy in geriatric patients is the high prevalence of polypharmacy (simultaneous use of five or more drugs), which may lead to higher incidence of drug interactions and therapeutic duplications [6, 7]. The complexity inherent in polypharmacy often leads to issues with patient compliance, resulting in a lack of adherence to the treatment that affects 40% to 75% of geriatric patients who are on such treatment regimens. This may be associated with confounding variables such as cognitive impairment, limited understanding of instructions, poor communication with health professionals, and increased likelihood of physical limitations [5].

Although there are several definitions for complexity of pharmacotherapy, there is general agreement that it does not rely on clinical, pharmacological, and demographic factors. Instead, the key influences are the dosage form, dose frequency, and the clarity of instructions for product usage [8]. The complexity of pharmacotherapy is composed of multiple features of the prescribed regimen including the number of different drugs in the treatment, number of doses of each individual drug per day, number of per-dose dosage units, total number of doses per day, and drug interactions with food [8–10].

In this context, the literature points out that pharmacotherapy reviews carried out by pharmacists may result in safer evaluations of how patients have been using their medications as well as the possible factors that make complex the pharmacotherapy of geriatric patients particularly [4, 11]. Elliott and colleagues [12] showed that pharmaceutical interventions reduced the complexity of pharmacotherapy index among geriatric patients in a specialized hospital from 6.7 to 4.7 points. The major impact of the intervention was in patients that were discharged, with an adjusted mean difference of 2.7 points, thus reducing the impact of hospitalization by 26.3% on the complexity of pharmacological treatment for those patients. Despite these reviews, there are few studies on the evaluation of medication complexity in Latin America.

### Aim of the study

This study aims to evaluate the complexity of pharmacotherapy in residents of three long-term care facilities (LTCF) in the state of Sergipe.

## Methods

### Design, location, and time of the study

A cross-sectional descriptive study was conducted for 12 months in three long-term care facilities, in the State of Sergipe, northeastern Brazil.

### Studied population

Pharmacotherapeutic records of all geriatric residents in the three long-term care facilities were evaluated. The sample population had the following attributes: men and women aged 65 years or older and using at least one prescription medicine.

### Data collection

The socio-demographic profile of the sample population was initially evaluated through the collection of data on age and gender. In addition, the number of medications used by the patients was verified. The data collected at the institutions were obtained from medical records, which were requested from the technical managers of the long-term care facilities. The BMI values were not collected, because the focus of the study was not to assess elderly patients diagnosed with chronic specific diseases. Also, mental/psychic status of patients was not obtained taking into consideration that the results would not influence the study, because the medicines used by the elderly were administered by the nursing staff.

### Evaluation of the pharmacotherapeutic profile

The characterization of the pharmacotherapeutic profile was evaluated with the following information: therapeutic duplicity was defined as “the use of more than one medicine from the same therapeutic class prescribed for the same clinical condition sorted according to the *Anatomical-Therapeutical-Chemical* classification system”; drug interactions were evaluated and classified according to the degree of severity using the *Micromedex®* database; potentially inappropriate medicines were analyzed according to the criteria of Beers (2012) which identifies 53 potentially inappropriate medications; and the presence of polypharmacy was defined as the multiple use of five or more medicines [13–18].

The total number of prescription medications was obtained for each patient, including both those of continuous or sporadic use. All prescription medications were included within the study; only homeopathic and manipulated medicines that did not present clear formulation were excluded.

### Assessment of the complexity of pharmacotherapy

To measure the complexity of pharmacotherapy, each medicine used was evaluated through the Medication Regimen Complexity Index (MRCI) instrument, translated, and validated for the Brazilian Portuguese by Melchiors et al. [10]. The MRCI has been applied only to medications prescribed by the doctor and/or indicated by the pharmacist evaluating the information contained on their label or prescription.

There are three sections in the MRCI instrument in which the following factors were assessed: section A (dosage forms present in the pharmacotherapy), section B (frequency of dose for each medicine in the pharmacotherapy), and section C (additional instructions for use, if applicable). Each section was completed in their respective order as per the instrument protocols, adding the score obtained in all three sections. Thus, the MRCI was obtained taking into account that the closer to 1.5, the lower the complexity of pharmacotherapy.

In this context, to analyze the relationship between the MRCI and the number of medicines, data were dichotomized to reflect the complexity of pharmacotherapy of the evaluated patients, as well as to relate with the analyzed variables [19, 20].

### Statistical analysis

All instruments were utilized and reviewed by the researcher and the results were recorded in Epi Info version 3.3 for Microsoft Windows with double entry. Data regarding socio-demographic profile, complexity of pharmacotherapy and pharmacotherapeutic profile of the patients were analyzed using descriptive statistics.

The Mantel-Haenszel chi-square test was used to evaluate the statistical significance of the association between socio-demographic and the pharmacotherapy data. Estimates of the prevalence of the number of medications according to the variables gender, age, complexity of pharmacotherapy, drug interaction, potentially inappropriate medications, therapeutic duplicity, and polypharmacy were calculated, in addition to the prevalence ratio, which is addressed as the measure of association. The associations between the dependent variables and each of the independent variables were investigated using bivariate logistic regression through calculations of the *Adjusted Odds Ratio*, 95% confidence intervals and *p*-values (*p* < 0.05). The MRCI values were analyzed as continuous variables. Data analysis was conducted using SPSS-PC version 13.0.

## Results

### Socio-demographic profile

Of the 132 patients in the LTCFs studied, seven (5.2%) did not use any medications of any kind and so were excluded. Thus, 125 patients who used at least one medicine composed the final sample, spanning the three LTCFs. The mean age of the sample was 81.8 years (±9.7, range 62–103), of which 66.4% were women.

### Pharmacotherapeutic profile

A high prevalence in the use of medications was observed in the three LCTFs, with approximately 95% of patients used at least one type of medication. From the analysis of 511 prescription medicines, 256 potential drug interactions were observed in 78 (62.4%) patients in the study sample.

One hundred and sixty-two (31.7%) potentially inappropriate medications were detected among the 125 analyzed patients. It is noteworthy that, in the defined sample, 92 (73.6%) patients used at least one medication considered inappropriate. Among the potentially inappropriate drugs most consumed, we highlight acetylsalicylic acid (28%), risperidone (10%), amitriptyline (8.5%), and thioridazine (8.5%). Table 1 shows the pharmacotherapeutic profile of the geriatric patients in all three LTCFs.

**Table 1:** Pharmacotherapeutic profile of the patients in the three long-term care facilities

Drug use prevalence (%)	94.7
Total consumption (*n*)	511
Female *n* (%)	327 (64)
Male *n* (%)	184 (36)
Medication per elderly (mean; SD)	4.0 ± 2.3
Minimum-maximum number	1–13
Patients who used <5 drugs *n* (%)	83 (66.4)
^a^Patients who used ≥5 medicines *n* (%	42 (33.6)
Medication present in the Rename 2010 (%)	62.4
Prescription drugs by generic name (%)	66.1
Medication complexity index (mean; SD)	15.1 ± 9.8
Minimum – Maximum	2–59
Potential drug interactions *n* (mean; SD)	256 (2.0 ± 3.0)
Minimum – Maximum	1–19
Potentially inappropriate medications for the elderly *n* (mean; SD)	162 (1.3 ± 1.1)
Minimum – Maximum	1–5
Therapeutic duplicity *n* (%)	45 (8.8)

Sergipe, Brazil, 2015

^a^Presence of polypharmacy

In this study, the patients that were in use of polypharmacy were more likely to have drug interactions (χ^2^ = 28.45; *p* < 0.0001), as well as using potentially inappropriate medications (χ^2^ = 17.04; *p* < 0.0001) and had therapeutic duplicity (χ^2^ = 70.7; *p* < 0.0001).

The bivariate analysis showed that there is no statistically significant association between age and the number of medicines used (χ^2^ = 0.35; *p* = 0.68), nor is gender difference related to the probability of presenting with polypharmacy (χ^2^ = 0.57; *p* = 0.57) (Table 2).

**Table 2:** Prevalence of polypharmacy in the studied population according to the variables: gender, age, drug interactions, potentially inappropriate medications for patients and therapeutic duplicity

Variable	Prevalence (%)	PR^(a)^	CI 95%	*p*-value
Gender				
Man	38.10	1.00		
Woman	31.33	0.82	(0.5–1.36)	0.28
Age				
≥ 80	31.58	0.86	(0.52–1.41)	0.34
< 80	36.73	1.00		
Potential drug interactions				
Yes	92.86	2.14	(1.65–2.78)	<0.0001
No	43.37	1.00		
Potentially inappropriate medications for the elderly				
Yes	97.62	1.53	(1.29–1.81)	<0.0001
No	63.86	1.00		
Therapeutic duplicity				
Yes	57.14	5.27	(2.7–10.3)	<0.0001
No	10.84	1.00		

Sergipe, Brazil, 2015

^a^Prevalence ratio

### Complexity of pharmacotherapy

The complexity of pharmacotherapy, measured by the MRCI, obtained a mean of 15.1 ± 9.8 (range 2–59).

Amongst those classified as having high levels of the complexity of pharmacotherapy, as defined above, an association with dose frequency was found with a mean of 5.5 ± 3.6; followed by additional instructions about the use of medicines averaging 4.9 (±3.7) and the dosage form averaging 4.6 (±3.0). This revealed a disproportion between the sections of the MRCI (Table 3).

**Table 3:** Complexity of pharmacotherapy of the elderly related to the number of medicines used according to each section of the MRCI

*n*	MRCI (mean)	Section A (*n*)	Section B (*n*)	Section C (*n*)	Number of medicines
15	3.9	08	04	03	01
20	7.0	10	06	04	02
18	10.4	02	08	08	03
30	14.3	05	12	13	04
09	18.1	02	03	04	05
15	21.8	02	10	03	06
11	27.0	03	04	04	07
03	30.3	00	00	03	08
01	44.0	01	00	00	10
01	43.0	00	01	00	11
01	49.0	00	00	01	12
01	59.0	00	01	00	13
Total *n* (%)	-	33 (26.4)	49 (39.2)	43 (34.4)	-
Mean (SD)	15.1 (±9.8)	4.6 (±3.0)	5.5 (±3.6)	4.9 (±3.7)	-

*N* represents the number of the elderly included in the study (*n* = 125). Sergipe, Brazil, 2015

Section A: dosage forms present in pharmacotherapy

Section B: dose frequency for each medication of pharmacotherapy

Section C: additional instructions, if present in the medication

The bivariate analysis showed a significant difference between the increased polypharmacy and the higher MRCI (χ^2^ = 77.93; *p* < 0.0001). Furthermore, our study found that the patients who had a high MRCI were more likely to have drug interactions (χ^2^ = 40.1; *p* < 0.0001). The finding was similar for the chances of using potentially inappropriate medications (χ^2^ = 15,8; *p* < 0.0001) and therapeutic duplicity (χ^2^ = 29.0; *p* < 0.0001).

The bivariate analysis also showed that for age there is no statistically significant association (χ^2^ = 0.95; *p* = 0.42). Moreover, the analysis pointed out that gender difference is not related to the probability of high MRCI (χ^2^ = 0.20; *p* = 0.79) (Table 4).

**Table 4:** MRCI prevalence in the studied population according to the variables: gender, age, polypharmacy, potential drug interactions, potentially inappropriate medications for patients and therapeutic duplicity

Variable	Prevalence (%)	PR^(a)^	CI 95%	*p*-value
Gender				
Man	47.62	1.00		
Woman	43.37	0.91	(0.91–0.39)	0.39
Age				
≥ 80	56.14	0.87	(0.65–1.16)	0.21
< 80	64.71	1.00		
Polypharmacy				
Yes	100	5.93	(3.68–9.56)	<0.0001
No	16.87	1.00		
Potential drug interactions				
Yes	92.86	2.46	(1.8–3.37)	<0.0001
No	37.68	1.00		
Potentially inappropriate medications for the elderly				
Yes	92.86	1.49	(1.22–1.82)	<0.0001
No	62.32	1.00		
Therapeutic duplicity				
Yes	50	6.90	(2.85–16.70)	<0.0001
No	07.25	1.00		

Sergipe, Brazil, 2015

^a^Prevalence ratio

The bivariate logistic regression was used to confirm the association between the MRCI values and the related risk factors. Similar to the bivariate analysis, our study found that the elderly who had high MRCI were more likely to have polypharmacy (OR = 2.796, 95% CI 1.652;4.732), potential drug interactions (OR = 1.275, 95% CI 1.163;1.397), therapeutic duplicity (OR = 1.133, 95% CI 1.071;1.198), and to use inappropriate medications (OR = 1.220, 95% CI 1.111;1.339) (Table 5).

**Table 5:** Logistic regression: risk factors associated with the complexity of pharmacotherapy

Variables	Adjusted OR (95% CI)	*p*-value
Polypharmacy	2.796 (1.652–4.732)	<0.001
Potential drug interactions	1.275 (1.163–1.397)	<0.001
Potentially inappropriate medications for the elderly	1.220 (1.111–1.339)	<0.001
Therapeutic duplicity	1.133 (1.071–1.198)	<0.001

Sergipe, Brazil, 2015

## Discussion

The mean age being over 80 years and the higher proportion of women in the studied LTCFs in this study resemble other studies in Brazil [21, 22], as well as those conducted in other countries [2, 23, 24]. The prevalence of drug use in the geriatric population is high, averaging from two to five drugs, depending on the methodology used, with higher consumption among female patients [24]. The use of pharmaceuticals by geriatric female patients may be connected to other singular factors specific to the aging process such as biological, social, and cultural problems [17]. In this sense, it is suggested that pharmaceutical interventions should be aimed specifically at women’s health needs, including increased training of the healthcare staff regarding the pharmacotherapy of morbidities affecting the female population.

### Assessment of the pharmacotherapeutic profile

In terms of prevalence of the number of medicines being used, the present study did not exhibit significant differences compared to similar studies [25, 26]. The clinician responsible for each long-term care facility prescribed all medications and, in all three institutions, these were administered by a nursing professional in a similar way to the study by Advinha et al. [24], which adopted as criteria the existence of individual drug records, nursing and medical support. This may help in the transcription of the pharmacotherapeutic profile and the assessment of the MRCI by the pharmacist.

Regarding the occurrence of therapeutic duplicity, the psycholeptic drugs (N05) were the most commonly identified agent in events of redundancy. According to Aguiar et al. [27], the use of psycholeptic drugs in geriatric patients is a common practice. Although the prescription of such drugs is common in Brazil, lack of clinical evidence-based indicators makes this a questionable practice [28]. The literature confirmed that the American Diabetes Association expects the duplication of the use of medicines for diabetes (A10) [29]. Furthermore, studies show that the use of multiple drugs is required to control diabetes and other conditions associated with it [30, 31]. In this sense, the assessment of therapeutic duplicity should not be directly linked to the quality of pharmacotherapy, in which evaluations about the risk-benefit balance cannot be carried out concurrently.

Overall, this study identified significant potential drug interactions in most institutionalized geriatric patients. We suggest requiring the monitoring of the outcomes of treatments prescribed to analyze whether the pharmaceutical interventions prescribed by the physician were of positive clinical significance. The literature confirms a positive association between the number of medicines and the presence of drug interactions pointing to polypharmacy as a precipitating factor of interactions and high mortality rates [32, 33].

Tarantino et al. [39] states that during the aging process there is a decrease in the overall hepatic metabolism of many drugs through the CYP enzyme system, and physiological changes from aging may increase the risk of drug-induced liver injury (DILI). Thus, polypharmacy and the presence of multiple diseases are considered factors that may increase the risk of drug interactions, as well as may cause exponential increase of drug-induced liver injury [34]. Studies have shown that clinically significant interactions are present in the pharmacotherapy of most geriatric patients presenting polypharmacy [16, 35–37].

In this study, most geriatric patients used at least one potentially inappropriate medicine. This often presents greater risk than corresponding benefits among this age group because of the increased probability of the incidence of intolerable adverse drug reactions and/or drug interactions. This type of situation is not limited to emerging countries; it is also common in developed countries [38, 39]. This may demonstrate a lack of knowledge regarding potentially inappropriate medications for geriatric patients by clinicians, which may introduce unnecessary risks to institutionalized geriatric patents and, therefore, higher costs for the institution.

It must be emphasized that the majority of potentially inappropriate medications prescribed in the LTCFs were present in the national list of essential and available medicines in the Brazilian health system. As the original foundation of the essential medicines list is to serve all ages and demands 80% of the diseases, we highlight the need for the development of a medications list directed specifically towards the treatment of geriatric patients, ensuring a more appropriate, effective, and safe pharmacotherapy [27]. Moreover, the participation of the clinical pharmacist as a member of the multidisciplinary team can help guide the physician in making decisions about appropriate pharmacotherapy, and evaluate the risk-benefit that the use of potentially inappropriate drugs may provide to the pharmacotherapy of these patients [4, 39].

In a study conducted by Delgado Silveira et al. [40], pharmacist intervention integrated into the multidisciplinary team managing geriatric, multi-pathological patients achieved a significant 59.7% reduction in drug-related problems. For this, further studies should be conducted on the effects of pharmaceutical interventions in order to support this analysis. Another alternative would be to provide a list of inappropriate medications to prescribers delivering positive potential in terms of patient safety [41, 42].

In this study, a significant proportion of the sample presented an increased prevalence of polypharmacy when compared to the literature [16–18]. It is worth emphasizing that the number of prescription drugs can be considered a risk factor for possible side effects in this age group [17, 33, 42]. Therefore, more investment is necessary in carrying out interventions focusing on the reduction of polypharmacy, considering the risk of occurrence of clinically-important adverse drug reactions increases by 50% with the concurrent use of five medicines, and 100% in cases that seven or more medicines are used [7, 18]. The same study also identified that in 20% of such cases the polypharmacy may cause severe side effects.

### Assessment of the complexity of pharmacotherapy

Few studies in the literature have used the MRCI as a tool to perform the assessment of complexity related to the pharmacotherapy [7, 43]. In this study, the complexity of pharmacotherapy measured by the MRCI obtained a mean of 15.1 ± 9.8 points (range 2–59), which is consistent with similar complexity results shown by Melchiors et al. [10], and demonstrated a mean MRCI of 15.7 points (±8.3, range 4–45.5).

When assessing the MRCI, the higher levels of complexity were associated with dose frequency, apparently related to the prescriptions with multiple schedules and scattered doses. This result differs from the literature, whereby the correlation between the total proportion of the complexity index and its three sections (A, B and C) did not reflect in a direct proportion, as it was expected by the developers of the original instrument [8, 24]. According to George et al. [8], the higher complexity index is the direct result of the individual growth of each section. However, this proportionality is not mentioned by the authors who validated the MRCI for the Brazilian Portuguese [10]. Since few studies have used the MRCI to assess the complexity index, further investigations are needed to confirm that the complexity of pharmacotherapy is not related to the direct proportion of each section (A, B and C) in the MRCI, and may occur disproportionality among them.

The present study found a direct association between the number of medicines used and the MRCI, which is confirmed by other studies in the literature [24, 44]. Although the results pointed out this relationship, the decrease in polypharmacy cannot be considered as the only protection against a higher MRCI [4, 7, 44]. Factors such as drug interactions, potentially inappropriate medications, and therapeutic duplicity may also influence the complexity of pharmacotherapy. Such data were confirmed by the logistic regression analysis where an increase in the MRCI values were associated with a significant increase in the probability of risk of polypharmacy, drug interactions, potentially inappropriate medications for the elderly and therapeutic duplicity. Therefore, pharmaceutical interventions to reduce the complexity index should not focus only on reducing the number of medications used.

The association of drug use with pharmacokinetic and pharmacodynamic changes linked to the aging process creates conditions for the higher risk of side effects and drug interactions observed among geriatric patients [45]. According to the literature, characterizing and simplifying the complexity of pharmacotherapy is necessary as the aging process creates appropriate conditions for the high risk of side effects and potential drug interactions [24, 45]. As a result, the pharmacist should identify and take action to avoid or mitigate potential drug interactions and therapeutic duplicity in order to optimize the risk-benefit relationship that such drugs can provide for these patients [46].

Regarding the three sections of the MRCI, which demonstrated higher levels of complexity, further studies are needed to guide future pharmaceutical interventions. It is believed that this is one of the first studies on Latin America that evaluates the MRCI in institutionalized geriatric patients.

The present study brings together diverse variables that have been analyzed in an isolated way in other studies such as: drug interaction, potentially inappropriate medication for the elderly, therapeutic duplicity and polypharmacy. Such fact allows a broad analysis of the complexity of pharmacotherapy of elderly patients, which may help to identify necessary points that aim towards interventions to reduce the complexity of pharmacotherapy. One limitation of this study is related to the fact that we did not stratify the sample study, considering obesity and mental disorder, once it may have underestimated some of the results. Moreover, this study cannot be generalized for all elderly patients since it was realized in LTCFs.

## Conclusion

The present study assessed some factors that make complex the pharmacotherapy of geriatric patients. Although we highlight polypharmacy as a factor directly related to the complexity of pharmacotherapy, other indicators such as drug interactions, the use of potentially inappropriate medications, and therapeutic duplicity can also hamper the use of pharmacotherapy in this group. Thus, the assessment of the complexity of pharmacotherapy by a clinical pharmacist may improve the analysis of prescription patterns, preventing health risks and ensuring greater safety. Further studies should measure the impact of such clinical interventions by a pharmacist in reducing the MRCI in order to minimize the risk factors that make complex the pharmacotherapy of geriatric patients in assisted long-term care facilities.

## Abbreviations

LTCFs: Long Term Care Facilities
MRCI: Medication Regimen Complexity Index
